# Elastolytic Granulomatous Dermatoses: Toward a Unified Clinicopathological Concept

**DOI:** 10.1111/ijd.70289

**Published:** 2026-01-16

**Authors:** Rose Kathrin Caroline Moritz, Christiane Michl, Sonja Leson

**Affiliations:** ^1^ Department of Dermatology, Venereology, and Allergology, Charité‐Universitätsmedizin Berlin Freie Universität Berlin and Humboldt‐Universität Zu Berlin Berlin Germany

**Keywords:** actinic granuloma, AEGCG, elastophagocytic giant cell granuloma, granuloma annulare

1

In their recent manuscript published in the *International Journal of Dermatology*, Poddine et al. describe the response to upadacitinib treatment in a patient with annular elastophagocytic giant cell granuloma (AEGCG) [1]. They discuss the Janus kinase/signal transducer and activator of transcription (JAK/STAT) pathway as a potential therapeutic target in this disease.

The case report is relevant given that the current treatment options for these patients are often insufficient and clinical studies are lacking.

Here, we would like to offer a dermatopathological perspective on the topic and highlight some key discussion points.

As the authors outline, AEGCG is characterized by dermal aggregates of multinucleated giant cells displaying signs of elastophagocytosis within the annular border of the lesions. These mark an abrupt transition to the central area with complete loss of elastic fibers. The histopathological characteristics partially overlap with those of granuloma annulare. The presence of mucin deposits and palisading histiocytes in the dermis surrounding necrobiosis have been discussed as being typical for granuloma annulare and absent in AEGCG. However, there has been some discussion as to whether these variable findings are sufficient to establish AEGCG as an independent diagnosis.

The term AEGCG was first mentioned in 1979 by Hanke et al. who reported five cases and reviewed previously published cases of “actinic granuloma,” “Miescher's granuloma of the face,” and “necrobiosis lipoidica presenting on the face and scalp,” summarizing all these entities under the diagnostic category of AEGCG [2]. In the same year, Ackerman reviewed 30 cases with granulomatous inflammation, observing elastophagocytosis in granulomatous skin diseases in sun‐exposed skin. These included foreign body reactions, mycosis fungoides, granulomatous syphilis, and most frequently, granuloma annulare. In 20 cases reported by Limas et al. features of necrobiosis and mucin deposits were seen in conjunction with “typical” lesions of AEGCG, calling into question the independence of this condition [3, 4].

We would therefore like to emphasize that, to our knowledge there are no clear‐cut clinicopathological criteria to differentiate between actinic granuloma, actinic elastolytic granuloma, and granuloma annulare. It appears probable that they all belong to the same spectrum of diseases where elastophagocytosis, loss of elastic fibers, necrobiosis, palisading histiocytes, and mucin deposits are variable findings.

We recommend to be careful not to split up entities with overlapping histological and clinical criteria. Historical and somewhat artificial classifications, derived from small sample sizes and without consideration of molecular characteristics, need to be critically re‐evaluated. JAK inhibitors have been successful in the treatment of single patients and case series with disseminated granuloma annulare and actinic granuloma [5]. In the interest of the affected patients, adopting a broad disease definition based on molecular pathogenetic mechanisms may therefore be beneficial and facilitate the initiation of much‐needed clinical trials in this indication.

## Conflicts of Interest

The authors declare no conflicts of interest.

## Data Availability

The authors have nothing to report.
